# To Move or Not to Move: When and How Bacteria Suppress Flagellar Motility

**DOI:** 10.1111/mmi.70085

**Published:** 2026-06-13

**Authors:** Fatemeh Mohaghegh, Jacob Scadden, Matthew A. B. Baker

**Affiliations:** ^1^ School of Biotechnology and Biomolecular Science University of New South Wales Sydney New South Wales Australia

**Keywords:** bacterial motility, biofilm, cyclic‐di‐GMP, flagellar motor, motility cessation, motility regulation

## Abstract

Bacterial motility enables cells to migrate toward favourable environments and initiate surface colonisation. While motility can be lost through passive processes, such as shearing of filament, it frequently occurs as a tightly regulated response shaped by environmental and ecological conditions. Cessation of motility is often not only a passive consequence of environmental limitations but can also represent a tightly regulated and adaptive strategy that promotes bacterial survival, persistence and biofilm development. Stopping motility allows the bacterial cells to redirect energy and metabolic resources toward essential processes, including extracellular matrix production and stress tolerance. This transition facilitates niche adaptation through protective biofilm communities which enhance bacterial stability under environmental stressors such as nutrient limitation, antimicrobial agents and host immune defences. Collectively, motility cessation represents a regulated shift to protective and communal lifestyles, with implications for bacterial ecology and pathogenesis. Studies have shown that bacteria use diverse mechanisms and signals for flagellar suppression, highlighting the importance of motility regulation in bacterial adaptation to changing environments. In this review, we highlight various mechanisms governing motility cessation and discuss the functional and environmental reasons that suppress motility across bacterial species.

## Graphical abstract

Motility cessation in bacteria is a key regulatory strategy that provides multiple survival advantages including enhanced community cooperation, niche adaptation and evasion of host immune responses. This process is controlled by associated mechanisms such as post‐translational modifications and second messenger signalling that stabilise non‐motile bacterial attachment. This review aims to provide a comprehensive overview of the molecular mechanisms in motility suppression and the functional and environmental conditions that trigger the transition from motile to non‐motile states.

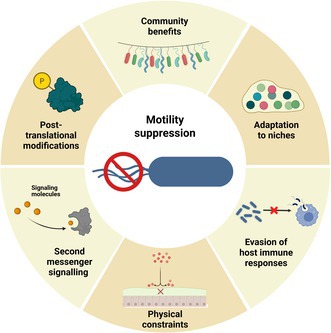

## 1 Introduction

Bacterial motility is a highly regulated process that allows cells to navigate their environments and colonise surfaces (Colin et al. 2021; Palma et al. 2022; Ramoneda et al. 2024). Motility supports some bacteria to move toward favourable environments and avoid harmful conditions to initiate surface attachment, leading to biofilm formation (Wolfson et al. 2020; Zheng et al. 2021; Benyoussef et al. 2022). The most widespread form is flagellar motility which involves a rotating, tail‐like organelle called a flagellum (Nakamura and Minamino 2019). It allows for swimming in liquid media and swarming across semi‐solid surfaces (Quelas et al. 2016; Kinosita et al. 2020). There are many alternative modes of bacterial motility, including twitching and gliding motility, which provide bacterial cells with motility when attached to surfaces (Varga et al. 2006; Antar et al. 2020). The motile to sessile transition is the process by which individual bacteria attach to a surface and transform into a multicellular community known as biofilm (O'Toole et al. 2000). This transition is significant because it represents a major lifestyle shift that allows bacteria to adapt rapidly to environmental changes such as nutrient fluctuations, oxygen levels and the presence of antimicrobial agents (Lebeaux and Ghigo 2012). Stopping movement enables bacteria to aggregate and produce an extracellular polymeric substance (EPS) matrix which provides a protective shield (Sharma et al. 2023). Moreover, by utilising specific signalling pathways and/or environmental cues, bacteria stop moving and initiate biofilm formation. Consequently, identifying and disrupting the molecular components that control motility and the sessile transition can help reduce motility and infection dissemination. In contrast, the ability to move is a fundamental adaptation that allows bacteria to move and exploit the heterogeneous and rapidly changing environments (Pessione 2021; Akahoshi and Bevins 2022). This capability also provides bacteria with several advantages that affect their survival (Berg 1975; Ottemann and Miller 1997; Cheong et al. 2015; Raina et al. 2019). For example, motile individual cells use motility to reach a surface and find appropriate sites for attachment. Later, subpopulations of cells may use motility to disperse from the biofilm to colonise new habitats (Boyd and Chakrabarty 1994; Millikan and Ruby 2004; Petrova and Sauer 2016). Additionally, in environments with constant fluid movement, such as the peristaltic flow of intestinal contents, non‐motile bacteria risk being washed away (Pereira and Berry 2017; Akahoshi and Bevins 2022). Motility allows bacteria to actively resist this flow, maintaining their position in favourable regions (Akahoshi and Bevins 2022). Despite its benefits, motility is also costly, with approximately 10% of energy spent on flagellum biosynthesis and motor function (Lynch and Marinov 2015; Schavemaker and Lynch 2022). For instance, motile marine bacteria consume a sizable fraction of their metabolic budget on motility (Taylor and Stocker 2012). Although loss of motility is passive in some environmental conditions, this review explores regulatory networks of motility arrest and discusses the evolutionary and ecological significance of stopping motility in bacteria.

## 2 Types of Flagella‐Mediated Motility and Their Regulation

### 2.1 Flagellar Motility

**FIGURE 1:**
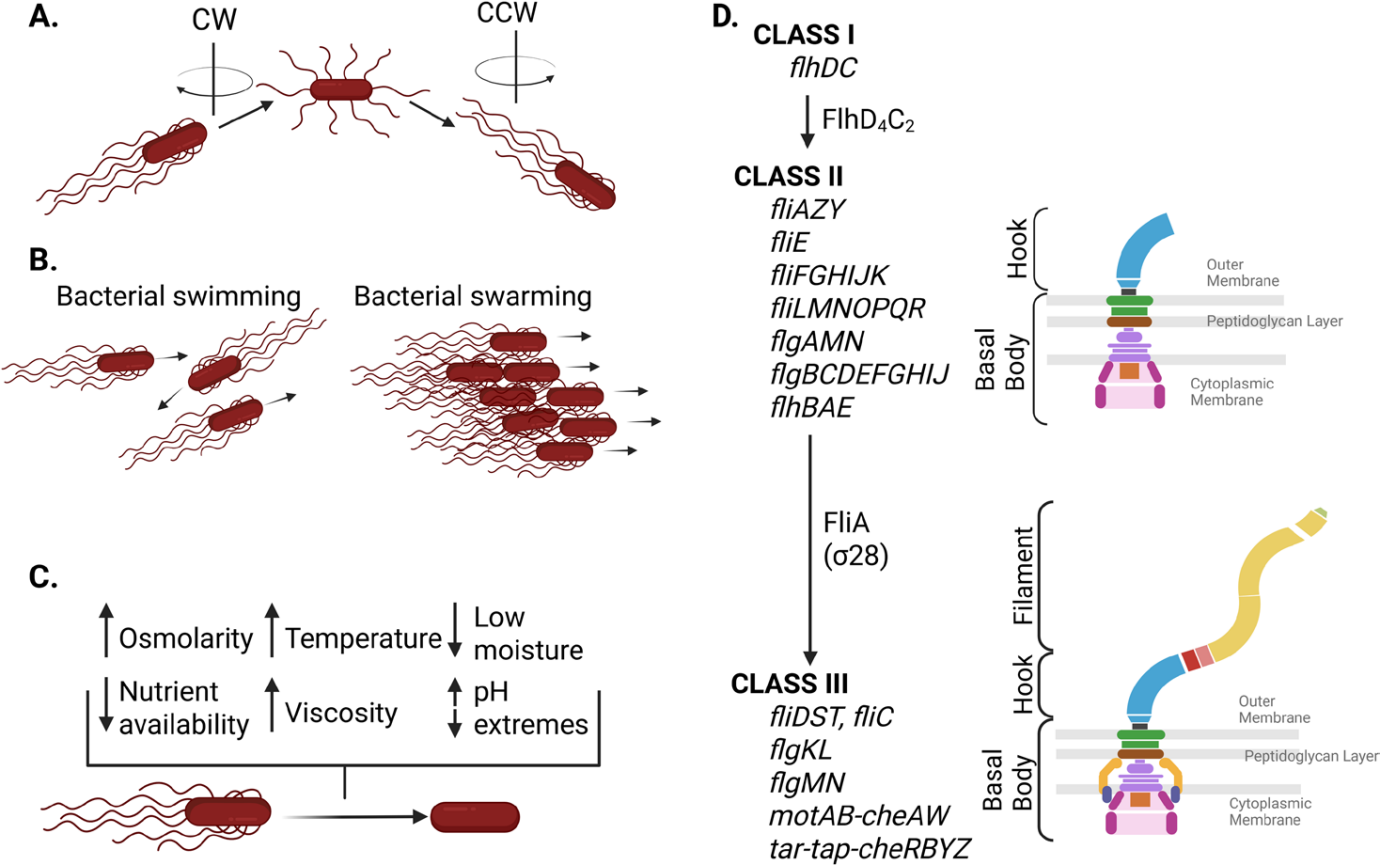
(A) Changes in swimming direction caused by switching of flagellar rotation from clockwise (CW) (tumbling) to counter‐clockwise (CCW) (running). (B) Bacteria swim freely in a planktonic state whereas bacteria will swarm when in semi‐solid media. (C) Bacteria will change from a motile to non‐motile state when osmolarity, temperature, nutrient availability and viscosity are increased, when moisture levels are decreased or at extremes of viable pH levels. (D) Flagella operon classes of *E. coli* , with the major transcriptional regular between each class, with a schematic of the flagellum structure being formed by expression of Class II and Class III genes.

Bacterial flagella are defined as thin, rigid, helical extracellular organelles, approximately 20 nm in diameter and extending 15–20 μm from the cell surface that enable flagellated bacteria to swim by rotation, with different arrangements (Imada 2017; Zhuang and Lo 2020). Flagellar arrangement varies across bacterial species and is highly correlated with their motility mechanisms (Grognot et al. 2023). For example, species with polar flagella can propel themselves forward or backward depending on flagellar rotation direction compared to those with peritrichous flagella that provide mostly unidirectional movement (Grognot et al. 2023). Additionally, the helical shape of flagella provides a highly efficient propellant system that enables bacteria to migrate rapidly in a chemical gradient (Purcell 1997; Xie et al. 2011; Kreutzberger et al. 2022). In *Escherichia coli* , the polar flagellar bundle facilitates fast swimming, characterised by a run‐and‐tumble pattern where the swimming direction is altered through switches in stator rotation between counter‐clockwise (running) and clockwise (tumbling) states (Figure 1A) (Kinosita and Sowa 2023). Swarming motility is described as the collective movement of bacterial populations across solid or semi‐solid media and is one of the main functions mediated through flagella (Figure 1B) (Quelas et al. 2016). This form of movement relies on the rotation of multiple flagella that enhances both surface adhesion and biofilm formation (Quelas et al. 2016). For example, in *Aeromonas* species, the lateral flagella required for swarming are also essential for adhering to epithelial cells (Gavín et al. 2002; Kirov et al. 2002). Energy costs involved with flagellar motility are high (Schavemaker and Lynch 2022). Therefore, optimising flagellar function is important not only for movement but also for energy conservation during reproductive and growth states (Huang et al. 2017; Schavemaker and Lynch 2022; Tătulea‐Codrean and Lauga 2024).

### 2.2 Environmental Factors That Affect Bacterial Motility

Bacteria regulate flagellar synthesis and activity in response to physical and chemical environmental conditions, increasing their adaptability and persistence in dynamic ecologies (Murakami et al. 2020; Grognot et al. 2023). Bacterial motility is highly influenced by various environmental factors such as nutrient availability, osmotic conditions and levels of stresses such as pH, temperature and oxygen availability (Figure 1C) (Rossi et al. 2018; Brauer et al. 2023). Nutrient availability is important for bacterial motility and behaviour. Under nutrient‐rich conditions, some bacteria exhibit enhanced motility, facilitating rapid colonisation of favourable niches (McCully et al. 2019). In contrast, nutrient limitation often suppresses motility, driving bacteria to adopt strategies that optimise resource utilisation such as forming aggregates to enhance nutrient acquisition (Wang and Or 2010; D'Souza et al. 2021). For example, polarly flagellated bacteria use various strategies to respond to nutrient gradients (Stocker 2012; Colin et al. 2021; Thormann et al. 2022; Antani et al. 2024). For instance, marine bacteria such as *Vibrio alginolyticus* and *Shewanella putrefaciens* show a run‐reverse‐flick behaviour, consisting of a forward run, a backward reversal and a flick that happens when the cell resumes forward swimming (Xie et al. 2011; Son et al. 2013). The efficiency of chemotaxis is enhanced through regulatory systems that integrate with the motility machinery to provide a more accurate response to changes in the environment (Parkinson 2003; Long et al. 2017). Osmoregulatory signals significantly impact bacterial motility. Hypo‐osmotic stress (low osmotic pressure) can also lead to flagellar assembly and function that subsequently impact swimming efficiencies (Ikeda et al. 2020). For instance, hypo‐osmotic stress induces flagellar biosynthesis and swimming motility in a significant portion of *Escherichia albertii* strains (Ikeda et al. 2020). Motile bacteria often show reduced motility when subjected to hyper‐osmotic situations until adaptations are made to the new osmotic condition (Li et al. 2019).

### 2.3 Signalling Pathways

The expression, assembly and regulation of flagella across various bacterial species are important for motility and associated functions. In *E. coli* , the flagellar regulon comprises over 50 genes across at least 17 operons and is modulated by several environmental cues, such as bacterial cell density (Chilcott and Hughes 2000; Sperandio et al. 2001). The flagellar regulon is categorised into three regulated, hierarchical transcriptional classes: class I, class II and class III (Figure 1D) (Kutsukake 1997; Chilcott and Hughes 2000). Class I genes code for the master regulators FlhDC which activates a transcriptional cascade of class II and class III flagellar operons. The *flhDC* operon encodes the FlhD and FlhC proteins which form a functional heterohexamer complex (FlhD_4_C_2_) (Wang et al. 2006; Lee et al. 2011). This complex acts as the master transcriptional activator required for the expression of all subsequent flagellar genes (Liu and Matsumura 1994; Soutourina et al. 1999). This operon integrates various environmental and metabolic signals via global regulators such as QseBC (quorum sensing) and CRP (catabolite repression protein) (Soutourina et al. 1999; Sperandio et al. 2002; Lee et al. 2011). Class II operon is directly activated by the FlhDC complex and primarily encodes components necessary for the structure and assembly of the hook‐basal body and transcriptional regulators FlgM and FliA (Liu and Matsumura 1994; Chilcott and Hughes 2000). The *fliA* gene encodes *σ* ^28^ which is required for the transcription of Class III genes. FlgM (anti‐ *σ* ^28^ factor) binds to *σ* ^28^ and prevents the transcription of class III promoters until the flagellar hook‐basal body complex is completed (Liu and Matsumura 1994, 1996; Lee et al. 2011). Class III promoters are activated by *σ* ^28^, encoding the final components of the flagellum such as flagellin (Chevance and Hughes 2008; Lee et al. 2011). It also includes genes for the motor torque generators (*motA*, *motB*) and the complete chemotaxis system (*che* genes and chemoreceptors) (Chilcott and Hughes 2000). Bacterial motility is controlled by multiple signalling pathways including chemotaxis and two‐component systems (Wadhams and Armitage 2004; Baker et al. 2006).

### 2.4 Two‐Component Systems

Two‐component systems (TCSs) are a widespread regulatory mechanism that allows bacteria to detect and respond to environmental or internal changes (Stock et al. 2000; Prüß 2017). It consists of two primary proteins: a sensor histidine kinase and a response regulator. When the sensor kinase detects a specific signal, it becomes autophosphorylated and transfers this phosphate group to its corresponding response regulator, activating it to regulate gene expression (Stock et al. 2000). The FlgRS TCS is a primary regulator of flagellar biogenesis in *Epsilonproteobacteria*, such as *Helicobacter pylori* and *Campylobacter jejuni* (Wösten et al. 2004; Kao et al. 2016; Kreling et al. 2020). This system is responsible for the transcriptional activation of class II flagellar genes, which are dependent on the alternative sigma factor *σ* ^54^ (RpoN). This system consists of FlgS, a cytoplasmic histidine sensor kinase, and FlgR, the corresponding response regulator that activates *σ* ^54^‐dependent transcription (Tsang and Hoover 2015; Kao et al. 2016). In *Enterobacteriaceae*, such as *E. coli* , the main TCS is QseBC. This system is encoded by an operon where the *qseC* gene encodes the histidine sensor kinase and the *qseB* gene encodes the response regulator (Sperandio et al. 2002). TCSs are typically activated through a phosphorelay cascade and acts as a transcriptional regulator of flagellar genes by activating the transcription of master flagellar regulator FlhDC (Table 1) (Stock et al. 2000; Sperandio et al. 2002). Additionally, chemotaxis has a primary role in bacterial movement as a mechanism to facilitate directed movement in response to chemical gradients. The process is mediated by sensor proteins that bind to chemical stimuli and initiate a complex signalling cascade, leading to changes in direction and activity in bacterial motility (Parkinson 2003; Sourjik and Wingreen 2012).

**TABLE 1:** Comparative summary of bacterial two‐component and phosphorelay systems.

System	Organism	Sensor component	Response regulator	Sigma factor(s)	Regulation of flagellar motility	References
FlgS/FlgR	*C. jejuni*	FlgS	FlgR	*σ* ^54^ (RpoN), indirect *σ* ^28^ (FliA)	Monitor formation of the flagellar type III secretion system, the MS ring and rotor structures; activate expression of *σ* ^54^‐dependent flagellar genes	Wösten et al. (2004)
FleS/FleR	*Pseudomons aeruginosa*	FleS	FleR	*σ* ^54^ (RpoN)	Major regulator of motility, flagellar biosynthesis, chemotaxis and biofilm formation; FleR activates transcription of multiple flagellar and pilus genes (e.g., *flgB*, *flgC*, *flgD*, *flgE*, *flgF*, *fliC*, *fliK* and *fliL*)	Zhou et al. (2021, 2022)
EnvZ/OmpR	*E. coli*	EnvZ	OmpR	*σ* ^70^ (direct)	Regulate flagellar gene expression by modulating the FlhDC master regulator	Shin and Park (1995) and Kenney and Anand (2020)
Rcs Phosphorelay	Enterobacteriaceae (e.g., *E. coli* , *Salmonella*)	RcsC → RcsD	RcsB (± RcsA)	*σ* ^70^, *σ*ˢ (RpoS)	Repress flagellar motility primarily by directly inhibiting *flhDC* transcription; promote a transition to a sessile, biofilm‐forming state, despite limited activation of some downstream flagellar genes (e.g., *fliPQR*)	Wang et al. (2007) and Fàbrega and Vila (2013)
FlrA/FlrB/FlrC	*Vibrio cholerae*	FlrB	FlrC (response regulator) and FlrA as upstream *σ* ^54^‐dependent activator	*σ* ^54^ (RpoN)	FlrA activates Class II genes (*flrB*, *flrC*) for polar flagellum biosynthesis; FlrB phosphorylates FlrC to activate the transcription of class III flagellar genes (e.g., *flgH*, *cheR*, *fliD*, *fliS*)	Syed et al. (2009) and Luo et al. (2016)

Bacterial motility is mainly driven by flagella, enabling swimming, swarming and surface attachment, and is regulated by environmental cues and signalling pathways such as chemotaxis, TCSs and cyclic‐di‐GMP. These systems regulate the transition between motile and sessile states in response to environmental changes. In addition to these regulatory systems, bacteria use multiple mechanisms to stop motility including gene regulation, post‐translational modification, flagella ejection and physical constraints that inhibit flagellar function.

## 3 Mechanisms of Motility Cessation and Transition to Sessility

### 3.1 Gene Regulation Reducing Motility

The downregulation of flagellar genes enables bacteria to adapt to their environments and facilitate processes such as colonisation, pathogenicity and survival (Table 2) (Spöring et al. 2018). In various bacterial species, flagellar genes are typically organised in operons and controlled by master regulators, such as FleQ in *Pseudomonas*, which mediate the expression of several flagellar genes and operons essential for motility (Dasgupta et al. 2003). Sigma factors, particularly sigma factor *σ* ^54^ (also known as RpoN), have been consistently linked to flagellar synthesis and motility mechanisms across various bacterial species (Tsang and Hoover 2014). For example, in *Vibrio cholerae* , FlrA is classified as a *σ*54‐dependent activator (Srivastava et al. 2013; Tsang and Hoover 2014). It sits at the top of a complex regulatory hierarchy and controls the expression of various classes of flagellar genes required for motility (Syed et al. 2009). They also enable bacteria to selectively transcribe specific genes in response to various environmental conditions. In *Clostridium difficile*, sigma factor D (*σ*D) plays an important role in the expression of flagellar and virulence‐associated genes found within the pathogenicity locus (PaLoc) (McKee et al. 2013; Anjuwon‐Foster and Tamayo 2017). Additionally, motility regulation in bacteria involves the identification of diverse transcriptional and post‐transcriptional mechanisms that fine‐tune flagellar gene expression. For instance, in *Campylobacter jejuni* , small RNAs (sRNAs) work alongside sigma factors to modulate flagellar synthesis in response to environmental changes such as nutrient or iron availability (König et al. 2024). In *Salmonella Typhimurium* , the transcriptional regulator AsiR directly binds to the *flhDC* promoter, activating the transcription of other downstream flagellar genes (Ma et al. 2021).

**TABLE 2:** Comparative overview of flagellar regulatory mechanisms in bacteria.

	Organisms	Main regulatory components	Regulatory mechanism/function	References
Sigma factors and master regulators	*Vibrio cholerae*	FlrA (*σ* ^54^‐dependent activator)	Activates *flrBC*, *flgK* and class III promoter	Prouty et al. (2001), Syed et al. (2009) and Li et al. (2022)
	*Pseudomonas aeruginosa* *Pseudomonas putida*	FleQ (*σ* ^54^‐dependent master regulator)	Activates flagella basal body assembly operons Regulated by FleN/FlhG, inhibiting FleQ ATPase activity to prevent excess flagella	Dasgupta et al. (2003), Nie et al. (2017), Chanchal et al. (2021) and Leal‐Morales et al. (2022)
	*Bacillus subtilis* *Salmonella enterica*	FliA (*σ* ^28^) and FlgM (anti‐*σ* factor)	FlgM secretion signals hook completion and releases *σ*28 to activate (class III) flagellar genes	Chilcott and Hughes (2000), Chevance and Hughes (2008) and Calvo and Kearns (2015)
	*Clostridium difficile*	*σ*D (alternative sigma factor)	Controls both flagellar and virulence genes (PaLoc) Links to motility to virulence	McKee et al. (2013) and Anjuwon‐Foster and Tamayo (2017)
	*Xanthomonas oryzae pv. oryzae (Xoo)*	RpoN2 (alternative *σ* ^54^)	Regulates motility and flagellar gene expression	Tian et al. (2015)
Transcriptional and post‐transcriptional regulators	*Campylobacter jejuni*	sRNAs (FlmE and FlmR)	Regulate stability and translation of flagellar mRNAs Modulate flagellar size and motility	König et al. (2024)
	*Salmonella Typhimurium*	AsiR (Acid signal‐induced regulator) FlgO (*σ* ^28^‐dependent sRNA)	Activates flagellar gene expression and enhances bacterial motility Negative regulatory effect on flagellar motility	Ma et al. (2021)
	*Escherichia coli*	LrhA FliX (*σ* ^28^‐dependent sRNA) MotR (*σ* ^28^‐dependent sRNA) ArcZ, OmrA, OmrB and OxyS	Transcriptional repressor of *flhDC* transcription Reduces flagella number and motility Promotes flagellar synthesis Repress the translation of *flhDC* mRNA	Melamed et al. (2023)

### 3.2 Second Messenger Signalling in Motility Down‐Regulation

**FIGURE 2:**
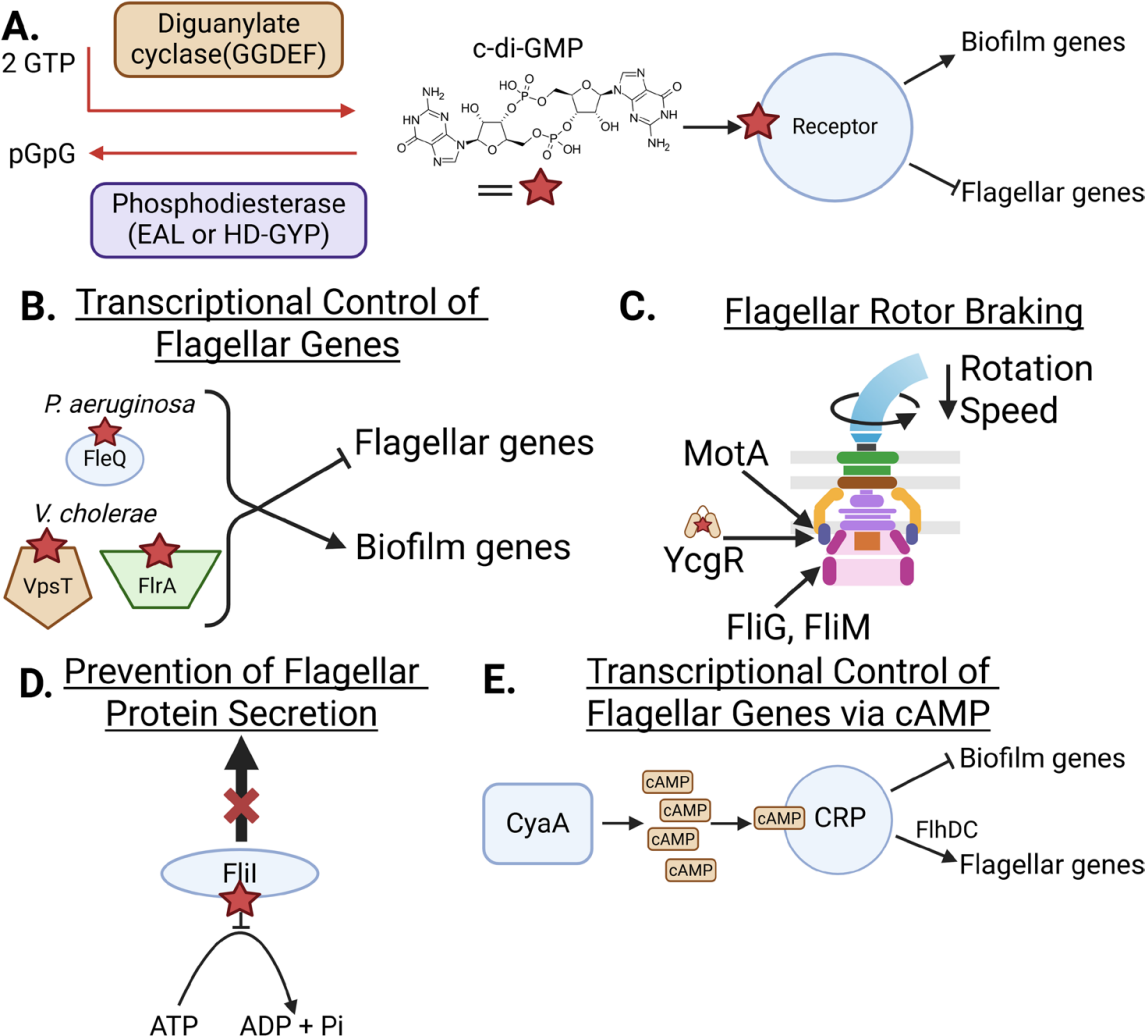
(A) Components of c‐di‐GMP signalling networks. At the cellular level, the concentration of c‐di‐GMP is regulated by diguanylate cyclases containing a GGDEF catalytic domain and phosphodiesterases, which possess either an EAL or HD‐GYP catalytic domain. Internal and external signals are directly or indirectly modulated by the enzymes through accessory domains. (B) By interacting with various receptors, such as FleQ in *P. aeruginosa* and VspT and FlrA in *V. cholerae* , c‐di‐GMP plays a crucial role in inhibiting bacterial motility and promoting biofilm production. (C) Interaction of c‐di‐GMP with flagellar break protein, YcgR, which subsequently interacts with the stator units and reduces rotation speed of the flagellum. (D) c‐di‐GMP binding to FliI, preventing ATP binding which leads to prevention of flagellar protein export. Panels (A)–(D) are adapted from Chen et al. (2025). (E) The generation of cAMP by CyaA, which then interacts with CRP which can increase the expression of flagellar genes and increase motility. Adapted from Stella et al. (2008).

Bis‐(3′‐5′)‐cyclic dimeric guanosine monophosphate (c‐di‐GMP) is now broadly recognised as a conserved bacterial intracellular second messenger and a key regulator in bacterial transition from a motile to a sessile and subsequent biofilm lifestyle (Yang et al. 2011; Zorraquino et al. 2013). The concentration of this signalling molecule is regulated through the action of diguanylate cyclases (DGCs) and c‐di‐GMP‐specific phosphodiesterases (PDEs) (Figure 2A) (Chou and Galperin 2015). DGC activity depends on a conserved GGDEF domain, which catalyzes the conversion of two Guanosine Triphosphate (GTP) molecules into c‐di‐GMP, while PDE activity is associated with the EAL and HD‐GYP domains, which degrades c‐di‐GMP into linear 5′‐Phosphoguanylyl‐(3′,5′)‐guanosine (5′‐pGpG) or two GMP molecules (Zorraquino et al. 2013).

More recently this soluble molecule has been shown to play a highly significant role in controlling adhesion, motility, virulence and cell morphogenesis in diverse bacterial species by exerting control at the level of transcription, translation and posttranslational modifications (Boyd and O'Toole 2012). In *E. coli* , c‐di‐GMP modulates motility primarily through two characterised pathways: direct interaction with motor proteins and regulation of transcription factors involved in flagellar biosynthesis (Boehm et al. 2010). Similarly, in *P. aeruginosa*, elevated c‐di‐GMP levels promote binding of c‐di‐GMP to FleQ (central transcriptional regulator) which leads to activation of biofilm‐related operons (Figure 2B). This regulatory switch enables a rapid transition from a swimming state to a sessile biofilm lifestyle (Figure 2B) (Chen et al. 2025). Moreover, c‐di‐GMP binding to ATPase domain of FliI, a subunit of the export apparatus, could halt the export of extracellular subunits for flagellar assembly (Figure 2D) (Chen et al. 2025). *V. cholerae* uses a similar but more complex regulatory hierarchy involving multiple transcription factors such as FlrA and VpsT (Chen et al. 2025). In *Serratia marcescens* , the cyclic AMP (cAMP)‐dependent catabolite repression system (CRS) is a critical positive regulator of flagellum production and swimming motility (Stella et al. 2008). When bacteria encounter less favorable or limiting carbon sources, adenylate cyclase (encoded by *cyaA*) is activated, leading to increased cAMP levels. The formation of cAMP‐CRP complex induces the expression of *flhDC* operon, thereby promoting flagellar gene transcription (Figure 2E) (Kalivoda et al. 2008; Stella et al. 2008).

### 3.3 Flagellar Brake Proteins

YcgR (c‐di‐GMP‐binding protein) interacts with the flagellar motor components, particularly MotA, FliG and FliM proteins, serving as a brake that inhibits motor function and alters flagellar rotation dynamics (Figure 2C) (Boehm et al. 2010; Fang and Gomelsky 2010). This direct inhibition contributes to a reduction in motor speed and alters directional bias during chemotaxis, a phenomenon termed the ‘backstop brake’ mechanism (Paul et al. 2010). A similar regulatory strategy operates in *P. aeruginosa* where motility is modulated by the YcgR homologue FlgZ and MapZ (c‐di‐GMP‐binding adaptor protein) which respond to c‐di‐GMP levels and control flagellar motor activity (Ma et al. 2020). Elevated c‐di‐GMP levels may modulate the interaction between transcriptional regulators such as RssB and stress sigma factor *σ* ^S^, which contributes to the repression of flagellar gene expression and motility inhibition (Nieto et al. 2019). Similarly, in *Salmonella enterica* , the impact of c‐di‐GMP on motility is significant. High intracellular concentrations of c‐di‐GMP reduce flagellar function, primarily through the action of YcgR, while increasing cellulose synthesis through the BcsA protein, which is responsible for producing the main exopolysaccharide of the biofilm matrix (Zorraquino et al. 2013).

### 3.4 C‐di‐GMP Activated Gene Regulation

In *V. cholerae* , c‐di‐GMP represses flagellar motility by influencing the transcription of flagellar operons (Srivastava et al. 2013). One of the key c‐di‐GMP binding transcription factors involved in this process is VpsT which serves as a sensor for intracellular c‐di‐GMP levels. When c‐di‐GMP concentrations are high, VpsT represses flagellar motility and together with VpsR promotes the production of the extracellular polysaccharides that are characteristic of biofilm growth (Figure 2B) (Krasteva et al. 2010; Srivastava et al. 2013). PDEs that degrade c‐di‐GMP modulate its level in response to various internal and external stimuli such as quorum sensing and low temperature, serving as a regulatory mechanism that influences whether *V. cholerae* adopts a motile or sessile state (Townsley and Yildiz 2015; Conner et al. 2017; Fernandez et al. 2020).

### 3.5 Post‐Translational Modifications

Post‐translational modifications (PTMs) of proteins are crucial in bacterial adaptability and cell cycle control (Grangeasse et al. 2015). Most bacterial PTM mechanisms, including proteolysis and phosphorylation, enable the cells to use them as effective regulatory devices in various biological contexts such as motility modulation (Hughes et al. 2018).

#### 3.5.1 Proteolysis

Proteolysis as a significant form of PTM is irreversible and considerably impacts motility proteins. For instance, in *Bacillus subtilis* , SwrA is the master activator protein which is necessary for flagellar biosynthesis and motility. This motility regulator is tightly controlled by the LonA and SmiA proteases, modulating SwrA levels based on environmental conditions such as surface contact, thereby affecting flagellar density and consequently motility (Mukherjee et al. 2015; Hughes et al. 2018). This regulation is essential for maintaining an appropriate balance between motile and sessile states, as excessive SwrA can lead to hyperflagellation which is detrimental to cell viability in liquid environments (Mukherjee et al. 2015). Moreover, the Clp protease system not only controls swimming motility through degradation of the transcription factor ComK but also affects the stability of other regulators like DegU that is crucial for flagellar gene expression (Molière et al. 2016).

### 3.6 Flagella Ejection

The deactivation of flagella in different environments is diverse across different bacteria. The α‐proteobacterium *Caulobacter crescentus* actively ejects its single polar flagellum to build an adhesive stalk for surface adhesion, while γ‐proteobacteria eject their polar flagella at the base of the flagellar hook in response to nutrient depletion (Ferreira et al. 2019). Polar‐flagellated γ‐proteobacteria such as *Plesiomonas shigelloides* and *V. cholerae* have one or more polar flagella whose motors are powered by sodium ions. These γ‐proteobacteria show a mechanism where the ejection is triggered by starvation, which acts as a necessary and sufficient signal for the bacteria to enter a non‐motile state (Ferreira et al. 2019). For instance, *Vibrio* species eject their flagella when nutrients become scarce and leave a relic of the former flagellar motor in the outer membrane to prevent the leakage of cellular contents across the outer membrane and periplasm (Ferreira et al. 2019). Furthermore, specific proteins are required for the flagellar ejection process. In *Caulobacter crescentus*, flagellar ejection is triggered by the cell cycle‐dependent proteolysis of FliF, the MS ring protein that anchors the flagellum in the inner membrane (Jenal and Shapiro 1996; Aldridge and Jenal 1999). FliL is necessary for the efficient degradation of FliF during the transition from a motile to a non‐motile state and appears to function in the same regulatory pathway as the diguanylate cyclase PleD (Aldridge and Jenal 1999; Wolfe and Visick 2008). Together, these proteins contribute to the destabilisation of FliF to facilitate ejection (Wolfe and Visick 2008). In addition, FliL may act as a sensor that recognises surface contact. This sensing would then signal the cell to transition from a planktonic lifestyle to a surface‐attached state by inducing flagellar ejection (Wolfe and Visick 2008).

### 3.7 Physical Constraints Limiting Motility

The cessation of bacterial movement in physically constrained environments is mainly associated with surface attachment. This adhesion can be mediated by the mechanical forces triggered by flagellar activity that enable bacteria to overcome repulsive forces and attach effectively to surfaces, which is a crucial step for initiating biofilm production (Friedlander et al. 2015; Roux et al. 2018; Wolfson et al. 2020). In addition, the adhesive capacity of flagella is enhanced by the presence of specific adhesive organelles or surface components, such as pili and lipopolysaccharide, that help bacteria to interact with host tissues during infections (Friedlander et al. 2015; Horstmann et al. 2017). For example, when *E. coli* establishes effective adhesion, synthetic adhesins (SAs) signals are transduced by the bacterium to stop movement. Notably, the arrest of flagellum rotation during the transition between motile and surface‐attached multicellular communities is regulated by intracellular levels of the c‐di‐GMP second messenger (Fang and Gomelsky 2010; Piñero‐Lambea et al. 2015). *P. aeruginosa* uses both a single polar flagellum and type IV pili (T4P) to sense surfaces (Burrows 2012; Maier and Wong 2015). Upon encountering a surface, initial contact can be mediated by the flagellum. This sensing is extremely rapid and depends on the MotAB stator (Laventie et al. 2019; Schniederberend et al. 2019; Geiger et al. 2024). Interestingly, *P. aeruginosa* can sense surfaces even without a flagellar filament (FliC), suggesting that the membrane‐embedded motor components themselves are the primary sensors (Lele et al. 2013; Laventie and Jenal 2020). The T4P are dynamic appendages that extend and retract. When a pilus tethered to a surface retracts, it generates tensile forces, detected by chemosensory‐like signalling pathways (Pil‐Chp system) (Talà et al. 2019; Laventie and Jenal 2020; Geiger et al. 2024). In *V. cholerae* , surface exploration is driven by its flagellum and while mannose‐sensitive hemagglutinin (MSHA) pili contribute to the early attachment. These MSHA pili facilitate an initial ‘orbiting’ behavior close to the surface before the cells make an irreversible attachment (Utada et al. 2014; Jones et al. 2015).

The physical properties of surfaces significantly impact motility cessation. Rough or hydrated surfaces alter bacterial interactions, as factors such as the aqueous film thickness and surface topography affect flagellar motility. For instance, surface roughness can mechanically block the flagellar rotation and promote the transition to a sessile state (Tecon and Or 2016). Moreover, fluid flow generates shear forces that can physically disrupt the flagellar motor, leading to a stop in motility and promoting surface adherence. For example, in *P. aeruginosa* , adhesion to a surface under shear flow produces forces that bend the upstream‐facing flagellum around the cell body which prevents rotation (Palalay and Sanfilippo 2025).

Bacterial motility cessation is achieved through regulatory and structural mechanisms. Transcriptional repression of flagellar genes limits flagellar synthesis, while c‐di‐GMP signalling inhibits motor activity via proteins such as YcgR. Additionally, physical processes including flagellar ejection and physical constraints associated with surface attachment contribute to the loss of movement. However, motility cessation provides key adaptive advantages under specific environmental conditions. By transitioning from a motile to a sessile state, bacteria can conserve energy, improve surface attachment and promote cooperative community behaviours that enhance persistence and stability.

## 4 When Bacteria Stop Moving: Adaptive Advantages of Motility Cessation

### 4.1 Energy Conservation

Bacterial motility is one of the most resource‐demanding processes, consuming around 10% of a cell's metabolic resources for protein synthesis and metabolic energy (Honda et al. 2022; Schavemaker and Lynch 2022). The rotation of flagellar motors is powered by the ion‐motive force, primarily the proton motive force (PMF). This electrochemical gradient is used for ATP synthesis and growth processes. The relationship between these systems is highly dependent on the ecological context (Ni et al. 2020; Buckel 2021).

### 4.2 Environmental and Pathogenic Triggers

The cessation of motility is often a coordinated response to environmental transitions where survival or defense becomes the priority over movement (Kan et al. 2018). When a pathogen like *Xanthomonas campestris* contacts host tissues, motility becomes less essential. At this stage, the motility apparatus is subject to strict energy‐saving control to promote colonisation and survival within the host environment (Kan et al. 2018). In mature biofilms, motility genes are repressed to stabilise the multicellular aggregate and redirect energy toward biomass production and the synthesis of the EPS matrix (Guttenplan and Kearns 2013). Furthermore, upon entering starvation, some bacteria quickly stop swimming, actively brake their motors' rotation, and even release their flagella to preserve remaining energy reserves (Zhuang and Lo 2020; Honda et al. 2022).

### 4.3 Two‐Step Mechanism for Motility Cessation

To conserve energy, bacteria typically inhibit motility in two distinct stages that balance immediate needs with long‐term energy savings: functional inhibition and transcriptional repression. Functional inhibition is a temporary and rapid mechanism that operates at the motor level to stop or slow rotation without disrupting the flagellar structure (Guttenplan and Kearns 2013). For instance, in *Bacillus subtilis* , the protein EpsE acts as a clutch that directly binds to FliG. This protein–protein interaction separates the flagellar rotor from its power source, the MotA/MotB stator. In *E. coli* , the protein YcgR acts as a brake, reducing rotation speed and altering reversal frequency in response to high c‐di‐GMP levels (Guttenplan and Kearns 2013). For sustained energy savings, the cell must stop the costly production of new flagellar components, using transcriptional repression. Over time, flagellar gene transcription is inhibited, and existing flagella are diluted to extinction through subsequent growth within the biofilm. This shift ensures the cell does not waste energy on motility and the synthesis of the EPS matrix (Guttenplan and Kearns 2013).

### 4.4 Adaptation to Niches

**FIGURE 3:**
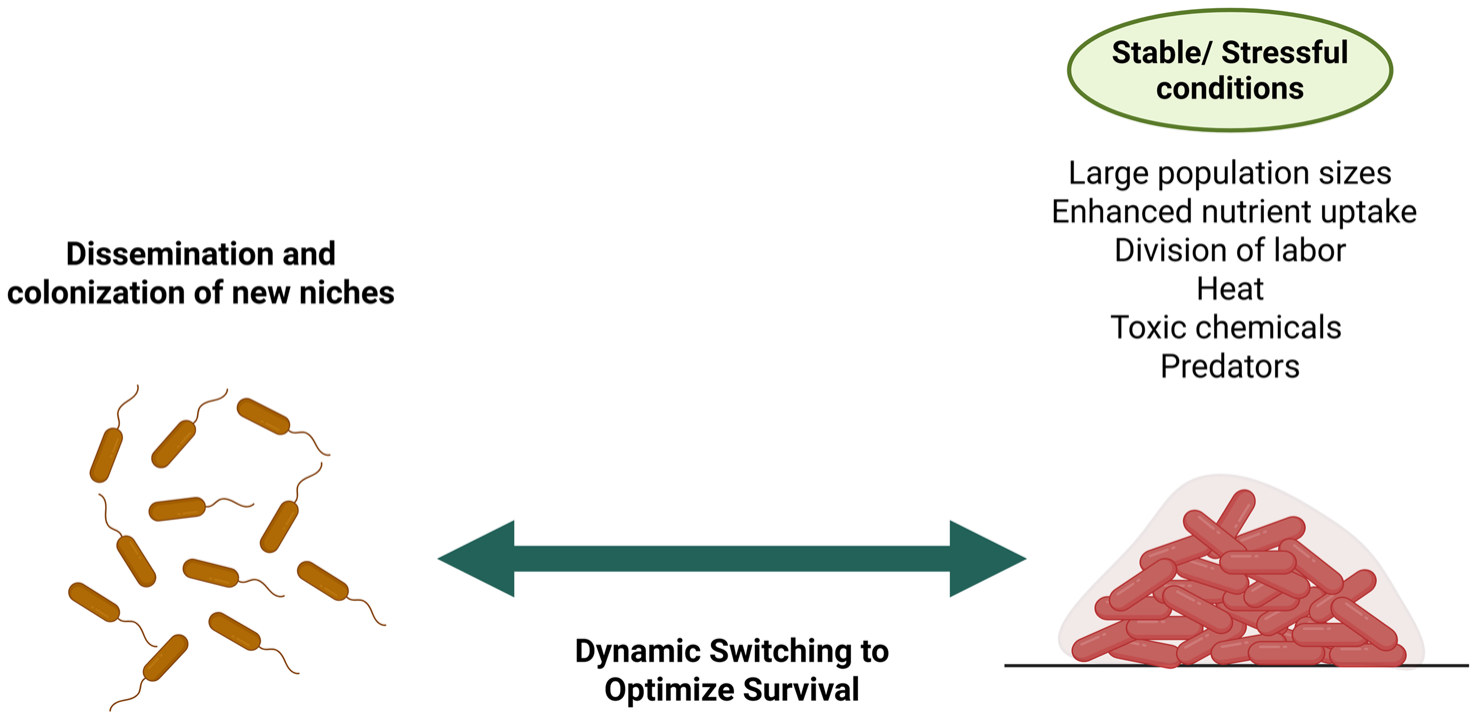
Schematic figure of the bidirectional switch between motile and sessile states, highlighting adaptive strategies. Motile cells with flagella enable dissemination and colonisation of new environments. Sessile biofilms support large populations with enhanced nutrient uptake in niches, facilitate division of labour for efficiency and provide persistence and protection against harsh conditions.

Bacteria adapt to diverse ecological niches by dynamically switching between sessile and motile lifestyles, a transition to optimise survival (Castiblanco and Sundin 2016; Bartolini et al. 2018). One example of this adaptive behaviour is the touch‐seed‐and‐go system which is a cooperative virulence and colonisation strategy used by bacteria such as *P. aeruginosa* (Laventie et al. 2019). In *P. aeruginosa* , this strategy is driven by the rapid modulation of the second messenger c‐di‐GMP (Laventie et al. 2019). Upon surface contact, c‐di‐GMP levels increase, leading to the effector protein FimW localising at the cell poles. Active FimW triggers the assembly of T4P, which serve as primary adhesins to facilitate the bacteria to the surface (Laventie et al. 2019). After surface attachment, bacteria undergo asymmetric division, driven by the unequal distribution of c‐di‐GMP (Laventie et al. 2019; Kreiling and Thormann 2023). This division produces two specialised cell types: the striker daughter cell inherits high c‐di‐GMP levels and remains committed to the surface, while the spreader mother cell inherits the flagellum and prevents surface attachment. These motile cells detach from the surface to colonise distant sites (Laventie et al. 2019). While motile states allow for the dissemination and colonisation of new habitats, the sessile state promotes persistence and protection in stable or stressful environments, respectively (Li et al. 2017; Penesyan et al. 2021). Formation of biofilms is a fundamental adaptive strategy that enables bacteria to survive harsh conditions such as heat, pH, toxic chemicals, nutrient availability and predators (Castiblanco and Sundin 2016; Ambreetha and Singh 2023). Moreover, in stable niches like the plant rhizosphere, healthy human gut or xylem vessels, sessility allows bacteria to develop large population sizes and enhances nutrient uptake (Castiblanco and Sundin 2016). Within these communities, bacteria benefit from a division of labor which allows the population as a whole to function more efficiently than individual cells (Bartolini et al. 2018). Conversely, motility allows planktonic cells to reach and colonise new environments (Barraud et al. 2015). However, in highly stressful environments like the cystic fibrosis lung, some bacteria like *Stenotrophomonas maltophilia* may eventually decrease their biofilm formation efficiency over years of chronic infection to disseminate and find new, ecologically favorable niches (Figure 3) (Pompilio et al. 2016). Some bacteria, such as *Burkholderia cenocepacia* , promote biofilm to withstand high doses of hydrogen peroxide which illustrates the protective role of biofilm formation (Peeters et al. 2010).

### 4.5 Evasion of Host Immune Responses

**FIGURE 4:**
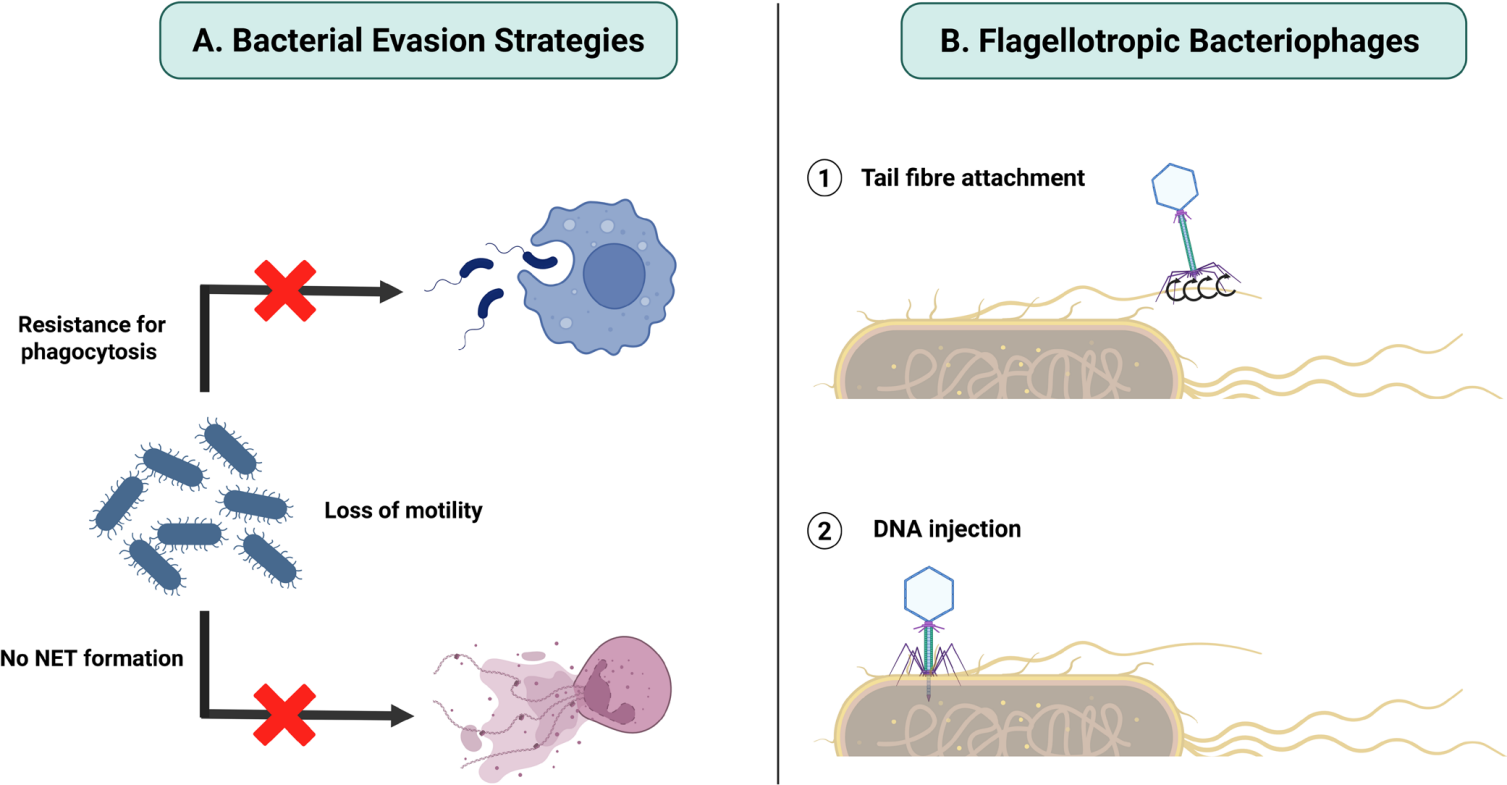
Schematic overview of bacterial evasion of host immunity and flagellotropic bacteriophage via flagella. (A) loss of motility in bacteria resists phagocytosis and immotile or motor‐deficient strains prevents Neutrophil Extracellular Traps (NET) release from neutrophils. (B) step 1 shows tail fibre attachment of a flagellotropic phage, wrapping around the helical flagellar filament of a bacterium. Step 2 illustrates the ‘nut and bolt’ mechanism, where rotation propels the phage to the cell surface for DNA injection. Adapted from Esteves and Scharf (2022).

The bacterial flagellum is a prominent organelle that serves as a key signal for the host immune system and bacteriophages (Terwagne et al. 2013; Esteves and Scharf 2022). Human immune cells detect flagellin, the primary subunit of the flagellum, through surface receptors like Toll‐like Receptor 5 (TLR5) and cytosolic receptors like NLR Family CARD Domain Containing 4 (NLRC4), which trigger inflammation, caspase‐1 activation and cytokine production (Terwagne et al. 2013). Many bacteria adopt a strategy of losing motility to evade some responses and survive in host environments. For example, in pathogens like *P. aeruginosa* , losing swimming motility results in a 10‐fold increase in resistance to phagocytosis by immune cells (Floyd et al. 2016). Flagellar motility, rather than the presence of the flagellum, is the primary factor that triggers the release of Neutrophil Extracellular Traps (NETs) from neutrophils. Consequently, immotile or flagellar motor‐deficient strains do not induce the formation of these lethal traps (Figure 4A) (Floyd et al. 2016). Furthermore, some bacteria, such as *Brucella*, use a stealth strategy by producing flagellin that does not activate TLR5 or by downregulating flagellar genes during infection to avoid triggering the NLRC4 inflammasome (Terwagne et al. 2013).

The immune system is not the only threat; flagellotropic (flagellum‐dependent) bacteriophages begin their infection cycle by targeting the flagellum as their primary receptor. For instance, *Salmonella* phage *χ* is considered the archetypal flagellotropic bacteriophage and belongs to the family *Siphoviridae* within the order *Caudovirales*, characterised by a long, non‐contractile tail. It employs a ‘nut and bolt’ mechanism in which a single tail fibre wraps around the flagellar filament and uses the flagellar rotation to reach the cell surface and continue infection (Figure 4B) (Esteves and Scharf 2022).

### 4.6 Community Benefits of Sessility

The cessation of motility is a reversible transition that allows individual bacteria to switch between motile and sessile states (Guttenplan and Kearns 2013). This transition promotes multispecies aggregation, metabolic cooperation and enhanced environmental resilience (Joshi et al. 2021; Schwartzman et al. 2022; Scarinci and Sourjik 2023). For a multispecies community to function, its members must remain in close physical proximity to establish stable interactions (Cordero and Datta 2016; Almeida et al. 2023). Slower dispersal rates promote coexistence by allowing species to co‐localise with beneficial facilitators while avoiding inhibitors or competitors. Moreover, unregulated motility can actually destabilise biofilm architecture, leading to flat, featureless structures rather than the complex, three‐dimensional shapes (Guttenplan and Kearns 2013; Lobanov et al. 2023). Specific cell–cell recognition and adhesion between different species minimise the distance between partners, which is critical for cell‐to‐cell sensing and metabolic exchange (Yao et al. 2022). For instance, conjugation occurs more often in biofilms than in planktonic states. In fact, the lack of movement and the presence of a stable matrix allow conjugative pili to remain intact, facilitating the spread of mobile genetic elements (MGEs) through direct cell‐to‐cell contact (Madsen et al. 2012).

### 4.7 Cooperation and Synergistic Functionality

The aggregate environment of a biofilm enables complex interactions that are impossible in a well‐mixed, planktonic state (Joshi et al. 2021). Aggregation allows the metabolic by‐products of one species to serve as nutrients by another (Joshi et al. 2021; Yao et al. 2022). For example, in oral biofilms, *Veillonella* species utilise lactic acid produced by *Streptococcus oralis* (Joshi et al. 2021). Motility cessation ensures that secreted beneficial products such as extracellular enzymes, biosurfactants and protective polysaccharides remain localised to the community rather than being diluted (Yao et al. 2022; Lobanov et al. 2023). Furthermore, microbial communities can achieve cross‐protection by absorbing or degrading toxic substances, such as antibiotics or metabolic waste, converting them into less harmful compounds for the benefit of nearby susceptible cells (Yao et al. 2022).

### 4.8 Benefits of Being Stationary Under Flow

**FIGURE 5:**
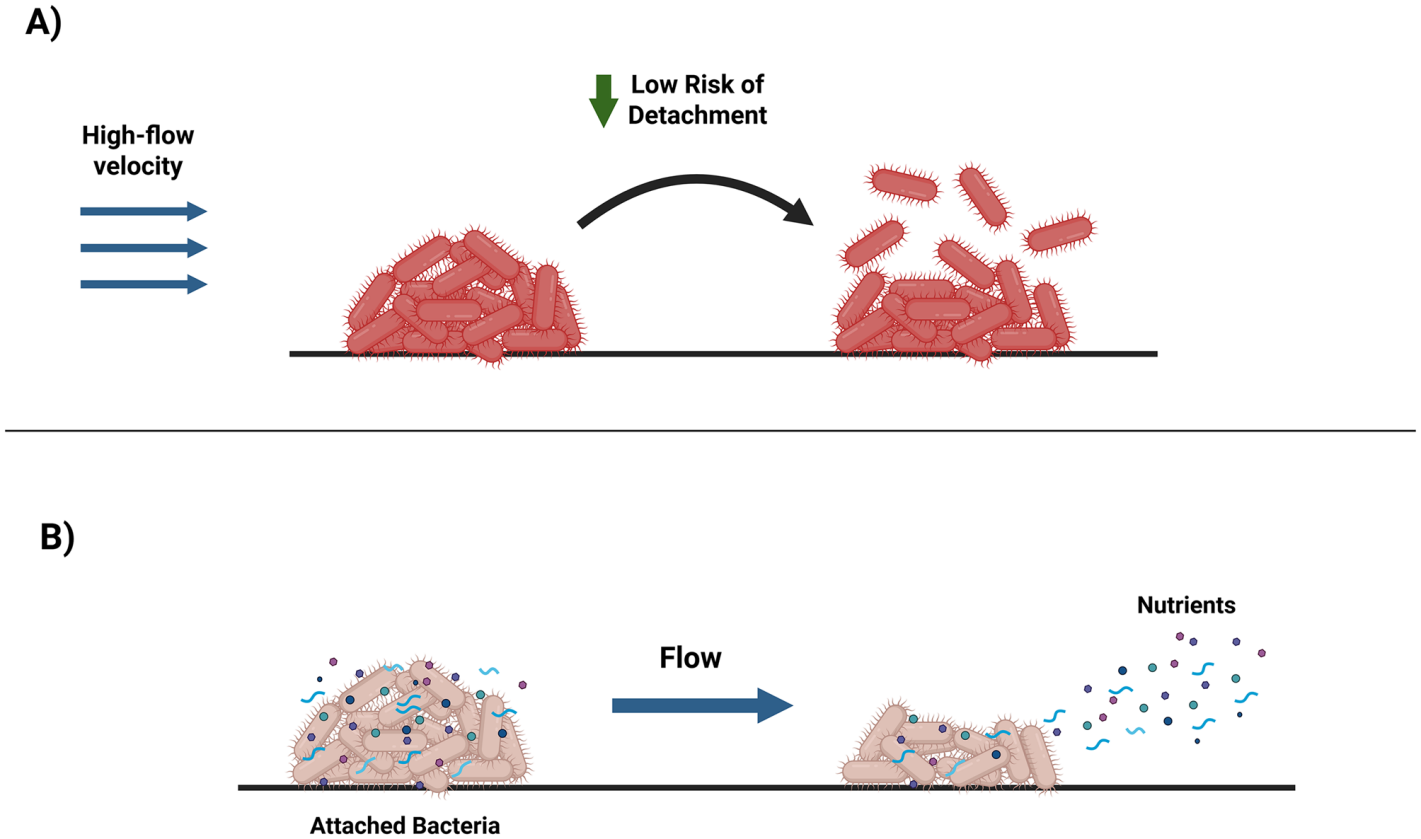
Advantages of transitioning to a sessile state in bacterial communities under fluid flow. (A) Mechanical stability against flow. In high‐velocity flow environments, aggregation into sessile clusters reduces the risk of detachment and enhances stability. (B) Metabolic benefits through nutrient capture. Surface‐attached bacteria secrete extracellular enzymes to digest substrates, releasing nutrients that are captured by producer cells before being dispersed by fluid flow. Adapted from Persat et al. (2015).

In an environment under flow, transitioning to a stationary or sessile state provides bacteria with critical physical and competitive advantages, ranging from mechanical stability to metabolic protection (Wong et al. 2021). In environments with high fluid velocity, the sessile state protects cells from mechanical shear forces that would otherwise detach and wash away individual cells (Figure 5A) (Scheuerman et al. 1998; Cheng et al. 2019). Furthermore, aggregation and attachment into biofilms increase local cell density and enhance cell–cell and cell‐surface interactions (Di Dio and Colin 2025). Some bacteria such as *C. crescentus* use the physics of flow to enhance their stationary populations. Their curved bodies experience hydrodynamic torque that rotates their free poles toward the surface, facilitating attachment and biofilm formation (Persat et al. 2015). In flow environments, bacteria can digest their substrate on which they reside by secreting extracellular enzymes, but liberated nutrients can freely diffuse away. By remaining stationary and attached, these producer cells can capture these nutrients before the flow carries them to distant cells that do not contribute to enzyme production (Figure 5B) (Persat et al. 2015). Although transitioning to a sessile state provides bacteria with certain advantages, motility under flow also confers ecological benefits (Padron et al. 2023). For instance, upstream swimming known as positive rheotaxis, which is a rapid and continuous motility mode, enables bacteria to move against shear forces, promoting retention near surfaces and facilitating migration toward favourable niches (Hill et al. 2007; Kaya and Koser 2012). In addition, moving with the flow helps bacteria reach areas with higher nutrient availability, where nutrients are delivered by fluid movement instead of relying on slow diffusion (Shen et al. 2024).

## 5 Conclusions

Bacterial motility shows a dynamic adaptation that facilitates exploration, colonisation and evasion in diverse environments, with the transition from motile state to sessile lifestyle, underscoring an important survival strategy in bacteria. As outlined in this review, flagellar motility is mediated by environmental cues like nutrient availability, signaling pathways including chemotaxis and TCSs, and second messengers such as c‐di‐GMP. Stopping motility is a highly regulated and adaptive strategy that supports bacterial survival, persistence and biofilm development. Mechanisms of motility suppression, including gene downregulation, flagella ejection and physical constraints, facilitate the planktonic‐to‐sessile transition. This shift promotes biofilm formation, thereby increasing resistance to antimicrobials and stressors. Stopping motility conserves energy by prioritising defense over movement, while it promotes niche adaptation, immune evasion, multispecies cooperation and stability in flow environments, preventing shear‐induced detachment.

Despite significant progress, several limitations remain in our understanding of motility arrest in bacteria. Mechanistically, motility can be regulated at multiple levels, which makes it often difficult to determine cause and effect within complex regulatory networks. Moreover, the timing of motility downregulation during infection or environmental changes remains poorly defined, limiting our understanding of its adaptive role. Finally, comprehensive comparative and evolutionary investigations are required to determine how motility cessation strategies are distributed and diversified across bacterial phyla. Future research should focus on comparative studies across species to identify species‐specific mechanisms of motility suppression and motor‐targeted inhibitors with broader regulatory strategies. Addressing these gaps will enable better models to predict how bacteria regulate motility in different environmental and host contexts.

## Author Contributions

**Fatemeh Mohaghegh:** conceptualisation, Visualisation, Writing – Original Draft Preparation, Writing – Review and Editing. **Jacob Scadden:** conceptualisation, Visualisation, Writing – Original Draft Preparation, Writing – Review and Editing. **Matthew A. B. Baker:** conceptualisation, Funding Acquisition, Supervision, Visualisation, Writing – Review and Editing.

## Funding

This work was supported by Australian Research Council (DP240100462).

## Disclosure

All figures were generated using BioRender.

## Ethics Statement

No human or animal subjects or materials were used in this review.

## Conflicts of Interest

The authors declare no conflicts of interest.

## Data Availability

Data sharing is not applicable to this article as no new data were created or analysed in this study.
